# 중환자실 간호사의 항생제 적정사용 관리 참여 영향요인: 횡단적 연구

**DOI:** 10.7586/jkbns.25.038

**Published:** 2025-08-26

**Authors:** Dogeon Jung, Nahyun Kim

**Affiliations:** 1 Infection control team, Kyungpook National University Hospital • Department of Nursing, Graduate School of Keimyung University, Daegu, Korea; 1 경북대학교병원 감염관리팀 • 계명대학교 대학원 간호학과; 2 College of Nursing, Keimyung University, Daegu, Korea; 2 계명대학교 간호대학

**Keywords:** Antimicrobial stewardship, Perception, Patient safety, Intensive care units, Nurses, 항생제 적정사용 관리, 인식, 환자안전, 중환자실, 간호사

## Abstract

**Purpose:**

This study aimed to identify personal and organizational factors influencing intensive care unit (ICU) nurses’ involvement in antimicrobial stewardship programs (ASPs).

**Methods:**

This descriptive cross-sectional study analyzed survey data for 159 ICU nurses during September 8 to October 23, 2023. Data analysis involved descriptive statistics, the t-test, analysis of variance, the Scheffé test, Pearson correlation coefficients, and multiple regression analysis.

**Results:**

The variables influencing participants’ ASP involvement were ASP perceptions (β = .35, *p* < .001), among personal factors, and patient safety culture (β = .18, *p* = .023) among organizational factors. The total explanatory power of these variables was 25.0% (F = 8.36, *p* < .001).

**Conclusion:**

The results of this study suggest that increasing ASP involvement requires improving ASP perceptions by clearly defining nurses’ roles in ASPs and providing professional ASP education. Proactive organizational efforts are also required to form a positive patient safety culture through the provision of educational programs.

## 서론

### 1. 연구의 필요성

항생제 내성은 10대 글로벌 보건 위협 요인 중 하나로 항생제 오용과 남용, 적절한 항생제 사용관리에 대한 인식과 교육의 부족으로 지속적으로 증가하고 있다[1]. 2022년 기준 국내 항생제 사용량(defined daily dose/1,000명/일)은 25.7로 경제협력개발기구(Organization for Economic Cooperation and Development) 평균인 18.9보다 약 36% 높은 수준이다[2]. 항생제 내성의 증가에 따라 세계보건기구(World Health Organization, WHO)는 2015년 세계보건총회에서 항생제 내성 대응을 위한 글로벌 행동 계획을 발표하였고, 항생제 내성 및 사용 감시 체계(global antimicrobial resistance surveillance system, GLASS)를 개발하여 모든 회원국에 국가 차원의 계획을 수립하고 참여하도록 제안하였다[1]. 이에 우리나라는 WHO와 연계한 국가 항균제 내성균 조사(Kor-GLASS) 운영[3]과 ‘제2기 국가 항생제 내성 관리 대책’을 통해 항생제 내성 감시와 항생제 적정사용 관리(antimicrobial stewardship program, ASP)를 도입하여 시행 중이다[4].

국가 항생제 내성 관리 대책에서는 항생제 내성 관리 전략을 크게 두 가지로 구분한다. 첫 번째는 ASP를 통해 항생제 오·남용을 예방하여 항생제 내성균 발생을 감소시키는 것이며, 두 번째는 이미 발생된 항생제 내성균이 추가적으로 확산되지 않도록 철저한 감염관리를 수행하는 것이다[4]. 항생제 관리(antimicrobial stewardship, AMS)는 항생제 사용을 체계적으로 감독하고 개선하는 광범위한 개념이며, ASP는 이를 실현하기 위한 구체적이고 조직적인 중재 프로그램이다[5,6]. ASP는 항생제 사용을 측정하고 개선하며 감시하는 일련의 활동을 통해 항생제의 종류, 용량, 투여 기간, 투여 경로 등의 적정성을 평가하고 개선함으로써 환자에게 최적의 항생제 치료를 제공하고 궁극적으로 환자안전을 증진시키기 위한 통합적 중재를 수행한다[5,6].

초기 ASP는 주로 전문적인 교육을 이수한 감염내과 의사와 약사가 주도해 왔지만 최근에는 의사, 약사, 간호사, 미생물학자 및 감염관리 전문가의 전문적인 지식을 활용한 다학제 기반 모델로 변화하고 있다[7-12]. 특히, 간호사는 의료기관 내에서 항생제를 가장 많이 다루는 직군 중 하나로, ASP의 성공적인 정착과 운영에 있어 간호사의 참여는 핵심적인 요소라 할 수 있다[7-12]. 간호사는 항생제 처방 확인 및 투여, 부작용 모니터링, 환자와 보호자에 대한 항생제 교육, 항생제와 관련된 다학제 의사소통 및 협력 등 ASP의 실질적인 운영과 효과 증대에 필수적인 역할을 수행한다[7,11,12]. 이러한 배경에서 국외에서는 간호사의 ASP 참여를 독려하고 있으며, 간호사의 역할에 대한 관심과 중요성이 점차 강조되고 있다[12]. 그러나 실제 임상에서는 간호사의 ASP 참여가 활발하지 않다는 보고가 이어지고 있으며, 그 원인으로 다양한 요인들이 지적된다[7-9,11-16]. 선행연구에 따르면 간호사의 ASP 참여를 저해하는 요인으로 ASP에 대한 인식 부족, 관련 지식 부족과 역할 인식 부족 같은 개인적 요인, 그리고 조직문화나 의료기관 차원의 제도적 지원 미흡과 같은 조직적 요인이 반복적으로 언급된다. 이 중에서도 ASP 인식과 조직문화는 간호사의 참여를 촉진할 수 있는 핵심적인 요인으로 지목되고 있다[7-9,11-16].

의료 전문가의 역할이 지속적으로 변화하는 상황에서 ASP에 대한 간호사의 인식과 태도를 탐색하는 것은 매우 중요하다[15]. 하지만 국내의 경우, ASP 인식과 참여에 관한 연구는 주로 의사나 약사를 중심으로 수행되었으며[17,18], 간호사를 대상으로 ASP 태도와 인식, 수행 등 일부 보고되고 있으나 매우 제한적인 수준이다[19,20]. 국내 간호사를 대상으로 한 연구에 따르면 국내 간호사의 약 20%만이 적절한 항생제를 사용하는 것에 대한 책임이 간호사에게 있다고 하여[19], 항생제 사용관리에 있어 간호사의 역할에 대한 인식이 부족하거나 업무 범위로 인식되지 않는 경향이 있는 것으로 보인다. 또한 국내 의료기관에서 활용 중인 ASP 지침에서도 간호사의 역할에 대한 구체적인 설명이 미흡하여[10,21] 간호사가 ASP에 효과적으로 참여하기 위한 기반이 부족한 실정이다.

간호사는 의료기관 내에서 가장 큰 비율을 차지하는 전문직 집단으로서[22], 안전한 항생제 사용관리에 중추적인 역할을 하고 있다[12]. 특히, 의료기관 구성원들의 환자안전문화에 대한 이해와 인식은 ASP 운영과 환자안전 향상에 필수적이므로[23] ASP의 효율성을 높이기 위해서는 간호사들이 인식하는 환자안전문화를 면밀히 살펴볼 필요가 있다[24]. 선행연구에서 간호사의 환자안전문화 인식 수준이 낮을수록 ASP 참여가 저조하게 나타났으며[7-9], 의료기관 구성원을 대상으로 환자안전문화 개선 프로그램을 적용한 결과 실제 항생제 처방률이 감소하는 등 구체적인 성과가 나타난 바 있다[25].

최근 WHO의 글로벌 행동 계획 권고에 따라, 국내에서도 ASP의 중요성이 강조되면서 상급종합병원과 종합병원을 중심으로 ASP 시범사업이 활발하게 시행되고 있다[4,10,21]. ASP는 국내 의료기관에서 점차 제도적으로 정착되어가는 단계에 진입했으나[10], 여전히 실무 현장에서의 적용은 제한적이며 다학제 간 협력이나 간호사의 실질적인 참여는 미흡한 수준으로 보고되고 있다[11-16]. 중환자실은 일반병동에 비해 환자의 중증도가 높아 다양한 항생제가 사용되고 있고, 항생제 내성균 발생률도 높기 때문에 중환자실 간호사의 ASP 참여가 더욱 시급한 실정이다[1,6]. 이에 ASP 참여에 영향을 미치는 개인적 요인으로 ASP 인식을, 조직적 요인으로 환자안전문화를 독립변수로 선정하였다. 이를 통해 간호사의 ASP 참여를 증진시키기 위한 효과적이고 구체적인 전략 수립에 근거자료를 제공하고자 한다.

### 2. 연구의 목적

본 연구의 목적은 중환자실 간호사를 대상으로 ASP 참여에 영향을 미치는 요인을 규명하는 것이다. 이를 위해 ASP 인식과 환자안전문화가 ASP 참여에 미치는 영향을 중심으로 다음과 같은 구체적인 연구목적을 설정하였다.

1) 대상자의 ASP 인식, 환자안전문화, ASP 참여 정도를 파악한다.

2) 대상자의 일반적 특성과 ASP 관련 특성에 따른 ASP 인식, 환자안전문화, ASP 참여 차이를 분석한다.

3) 대상자의 ASP 인식, 환자안전문화, ASP 참여 간 상관관계를 분석한다.

4) 대상자의 ASP 참여에 영향을 미치는 요인을 파악한다.

## 연구 방법

### 1. 연구 설계

본 연구는 대구광역시 소재 상급종합병원 경북대학교병원과 칠곡경북대학교병원 중환자실에 근무하는 간호사를 대상으로 ASP 참여에 영향을 미치는 요인을 파악하기 위한 서술적 조사연구이다.

### 2. 연구 대상

본 연구의 대상은 대구광역시 소재 상급종합병원 2곳의 중환자실에서 재직 중인 간호사로 선정기준에 부합하는 대상자를 편의표집하였다. 해당 병원들은 ASP 시범사업 이전부터 항생제 사용에 대한 세부 지침을 마련하고, 항균제관리 소위원회를 통해 사용 실태 및 내성 발현에 대한 분석을 실시한 후 그 결과를 직원들과 공유하고 있었다. 그러나 ASP 관련 교육은 시행되고 있지 않았다. 연구대상자 수는 G*Power 3.1.9.7 프로그램[26]을 이용하여 산출하였으며, 다중회귀분석을 위한 중간효과크기 .15, 유의수준 .05, 검정력 .85, 예측인자 14개를 고려하여 최소 148명이 산출되었다. 탈락률을 약 10%로 고려하여 총 164부의 설문지를 배부하였고, 이 중 응답이 불성실한 5부를 제외한 총 159명의 자료를 최종 분석에 사용하였다.

본 연구 대상자의 선정기준은 다음과 같다.

1) 중환자실 근무경력이 1년 이상인 간호사로 환자에게 항생제 투약간호를 제공하고 있는 간호자

2) 본 연구의 내용을 이해하고 참여에 동의한 자

### 3. 연구 도구

본 연구의 자료수집을 위해 구조화된 설문지를 사용하였다. 설문지는 일반적 특성, ASP 관련 특성, ASP 인식, ASP 참여, 환자안전문화에 대한 문항을 포함하고 있으며 모든 도구는 원저자에게 도구 사용에 대한 승인을 받은 후 사용하였다.

#### 1) 일반적 특성

일반적 특성은 성별, 연령, 결혼상태, 최종학력, 총 임상경력, 중환자실 임상경력, 현 근무부서, 직위의 총 8문항으로 구성하였다.

#### 2) ASP 관련 특성

ASP 관련 특성으로 건강보험심사평가원[27]의 항생제 적정사용을 위한 ASP 활성화 방안 연구에서 사용한 ASP에 대한 설문조사 문항 중 본 연구 목적에 맞게 재구성하여 사용하였다. 이중 항생제 내성 및 ASP 관련 지식 부분을 참고하여 항생제 내성 문제 인식 여부, ASP에 의한 항생제 사용감소 여부, ASP에 의한 환자 돌봄 및 안전 향상 여부 총 3문항으로 구성하였다.

#### 3) ASP 인식

본 연구에서 중환자실 간호사의 ASP 인식 정도를 측정하기 위해 Mustafa 등[13]이 개발한 도구를 사용하였다. 이 도구는 파키스탄 내 의료기관 간호사의 ASP 인식을 측정하기 위해 개발되었으며, 원문의 도구를 한국어와 영어 2개 국어를 사용하는 간호사 1인의 번역과 역번역의 과정을 거쳤다. 이후 대한항균요법학회 회장 1인, 대한항균요법학회 항생제 스튜어드십 연구회 회원인 감염내과 전문의 2인, 감염관리팀장 1인, 감염관리 전문간호사 1인, 감염관리 실무전문가 1인, 중환자 전문간호사 1인, 중환자실 수간호사 2인, 간호학 박사 1인으로 전문가 그룹을 구성하여 내용타당도(content validity index, CVI)를 측정하였다. 전체 CVI 평균은 0.94로 5개 문항 모두 CVI 0.8 이상이었다. 본 측정도구는 총 5개 문항으로 ‘전적으로 동의하지 않는다’ 1점, ‘전적으로 동의한다’ 5점의 Likert 척도로 구성되어 있다. 점수가 높을수록 ASP에 대한 인식이 높음을 의미하며, 원저자 연구와의 비교를 위해 수집된 점수를 100점 만점 척도로 환산하여 제시하였다. 본 도구의 신뢰도는 개발 당시 Cronbach’s α는 .71이었으며, 본 연구에서 Cronbach’s α는 .93이었다.

#### 4) ASP 참여

본 연구에서 중환자실 간호사의 ASP 참여 정도를 측정하기 위해 Mustafa 등[13]이 개발한 도구를 사용하였다. 이 도구는 ASP 인식 도구와 동일한 절차와 과정을 거쳤다. CVI 측정에 앞서 감염관리 전문간호사 자격증을 가진 간호학 교수 1인이 ‘문제 있는 처방에 개입한다’와 같이 국내 간호사의 직무상 오해의 소지가 있는 표현을 보다 적절한 의미로 수정하였다. 원 도구의 16개 문항 중 자료수집 대상 병원의 조직 편제상 일반간호사의 참여가 허용되지 않는 항생제 사용관리 위원회 참여, 의료기관 항생제 치료 가이드라인 개발 참여, 의료기관 내 항생제 종류의 관리 참여 문항이 CVI 0.8 미만으로 측정되어 제외하였고 전체 CVI 평균은 0.81이었다. 따라서 ASP 참여 측정도구는 총 13개 문항으로 ‘전혀 그렇지 않다’ 1점, ‘항상 그렇다’ 5점의 Likert 척도로 구성되어 있다. 점수가 높을수록 ASP에 대한 참여가 높음을 의미하며, 원저자 연구와의 비교를 위해 수집된 점수를 100점 만점 척도로 환산하여 제시하였다. 본 도구의 신뢰도는 개발 당시 Cronbach’s α는 .91이었으며, 본 연구에서 Cronbach’s α는 .87이었다.

#### 5) 환자안전문화

본 연구에서 중환자실 간호사의 환자안전문화를 측정하기 위해 Lee [28]가 개발한 환자안전문화 측정도구를 사용하였다. 환자안전문화 도구는 7개의 하부 영역을 포함하며, 여기에는 리더십, 환자안전 지식과 태도, 환자안전 정책과 절차, 환자안전 개선시스템, 환자안전 우선순위로 구성되어 있다. 본 측정도구는 총 35개 문항으로 구성되어 있으며, ‘전혀 그렇지 않다’ 1점에서 ‘매우 그렇다’ 5점까지의 Likert 5점 척도로서 점수가 높을수록 환자안전문화 정도가 높음을 의미한다. 본 도구의 신뢰도는 개발당시 Cronbach’s α는 .93 이었으며, 본 연구에서 Cronbach’s α는 .93 이었다.

### 4. 자료 수집

본 연구의 자료수집은 연구대상 병원의 중환자실에서 이루어졌다. 우선, 연구자가 직접 각 병원 간호부와 중환자실을 각각 방문하여 의료진에게 연구의 목적과 내용을 설명하고 연구 참여 협조를 구한 후 연구를 진행하였다. 자료수집은 ASP 시범사업 전인 2023년 9월 8일부터 10월 23일까지 이루어졌으며 설문지를 간호사실에 비치하여 자발적으로 참여할 수 있도록 하였다. 설문지 작성 후 밀봉하여 별도의 수거함에 넣도록 설명하고 설문지 비치 2주 후 연구자가 직접 수거하였다. 설문지 작성에는 15여분 소요되었으며 설문 응답을 완료한 대상자들에게는 소정의 답례품을 제공하였다.

### 5. 자료 분석

수집된 자료는 SPSS version 27.0 (IBM Corp., Armonk, NY, USA) 프로그램으로 분석하였고, 각 항목에 대한 구체적인 분석 방법은 다음과 같다.

1) 대상자의 일반적 특성과 ASP 관련 특성, ASP 인식, 환자안전문화, ASP 참여는 백분율과 평균, 표준편차를 산출하였으며, ASP 인식과 ASP 참여는 100점 척도로 환산하여 제시하였다.

2) 대상자의 일반적 특성과 ASP 관련 특성에 따른 ASP 인식, 환자안전문화, ASP 참여 차이는 Independent t-test와 analysis of variance로 검증하였으며, 사후검증은 Scheffé test로 분석하였다.

3) ASP 인식, 환자안전문화, ASP 참여의 간 상관관계는 Pearson correlation으로 분석하였다.

4) 대상자의 ASP 참여에 영향을 미치는 요인을 파악하기 위해 다중회귀분석을 이용하여 분석하였다.

### 6. 윤리적 고려

본 연구는 대구광역시 소재 경북대학교병원의 기관생명윤리위원회의 심의를 거쳐 연구 승인(KNUH 2023-07-033-006)을 받은 후 진행하였다. 자료수집 전 대상자들에게 연구의 목적과 절차, 연구 자료의 익명성 보장과 연구 비밀 유지, 연구 참여의 이익과 불이익에 대하여 설명하였으며, 언제든지 수집된 자료를 철회할 수 있음을 설명하였다. 충분한 설명을 들은 후에 자발적 의사로 연구에 참여하기로 한 대상에게 서면동의를 받고 연구를 진행하였다. 연구자 외 연구 참여 및 설문 내용을 알 수 없도록 동의서와 설문지는 별도로 밀봉되어 제출되도록 하였으며, 연구 참여 여부가 업무상 어떤 불이익도 없음을 설명하였다. 또한 연구 절차에 관한 문의 사항에 대하여 연락할 수 있도록 동의서에 연구자와 기관생명윤리위원회 연락처를 기재하였고, 동의서 및 설문 결과는 잠금장치가 설치되어 있는 보관 서랍에 보관되며, 3년간 보관 후 폐기할 것임을 안내하였다.

## 연구 결과

### 1. 대상자의 일반적 특성과 항생제 적정사용 관리 관련 특성

대상자는 총 159명으로 일반적 특성에서 여성이 139명(87.4%)으로 나타났고, 평균 29.57 ± 4.68세였으며 30세 미만이 96명(60.4%)이었다. 결혼상태의 경우 미혼이 121명(76.1%)으로 나타났고, 최종학력은 학사가 130명(81.8%)이었다. 임상 경력은 평균 77.4 ± 52.80개월로 나타났으며, ‘72개월 이상’이 78명(49.0%)으로 가장 많았다. 중환자실 경력은 평균 54.0 ± 32.80개월로, ‘36개월 이상 72개월 미만’이 81명(51.0%)으로 나타났다. 대상자의 현재 근무부서는 내과계 중환자실 89명(56.0%), 외과계 중환자실 70명(44.0%)이었다. 또한 직위별로는 일반간호사가 152명(95.6%)으로 가장 많았다.

ASP 관련 특성에서 항생제 내성 문제 인식의 경우 대상자 159명 모두 ‘예’라고 응답하였다. ASP에 의한 항생제 사용감소 여부를 묻는 질문에 ‘예’로 응답한 사람이 95명(59.7%)이었으며, ASP 적용에 의한 환자 돌봄 및 안전 향상 여부를 묻는 질문에 ‘예’로 응답한 사람이 143명(89.9%)으로 나타났다(Table 1).

### 2. 항생제 적정사용 관리 인식, 환자안전문화, 항생제 적정사용 관리 참여 정도

대상자의 ASP 인식은 78.79 ± 12.43점이었다. 5점 만점은 3.94 ± 0.62점이었으며, 최솟값은 2.00점, 최댓값은 5.00점으로 나타났다. 세부 문항 중에서는 '합리적인 항생제 처방 촉진'이 평균 4.03 ± 0.63점, '의료의 질 향상'이 평균 4.00 ± 0.72점으로 가장 높았고, '항생제 치료 비용 감소'는 평균 3.81 ± 0.76점으로 가장 낮았다. 환자안전문화는 5점 만점에 평균 3.66 ± 0.42점으로 나타났으며, 하위 항목으로 '환자안전 지식과 태도'가 평균 4.11 ± 0.46점으로 가장 높았고, '환자안전 우선순위'가 평균 3.34 ± 0.70점으로 가장 낮았다. ASP 참여는 60.67 ± 11.61점이었다. 5점 만점 평균은 3.03 ± 0.58점이었으며, 최솟값은 1.15점, 최댓값은 4.69점으로 나타났다. 세부 문항 중에서는 '항생제 투약 후 부작용에 대한 논의'가 평균 3.91 ± 0.84점, '처방된 항생제에 대한 검토'가 평균 3.85 ± 0.80점으로 가장 높았고, '중환자 항생제 치료 결정 참여'가 평균 2.03 ± 0.92점, '원내 항생제 사용량 확인'이 평균 2.16 ± 0.93점으로 가장 낮았다(Table 2).

### 3. 일반적 특성과 항생제 적정사용 관리 관련 특성에 따른 항생제 적정사용 관리 참여 차이

일반적 특성에서 ASP 인식의 경우 결혼 상태(t = −2.84, *p* = .003)에서 유의한 차이가 나타났다. 환자안전문화의 경우 성별(t = 2.97, *p* = .003), 결혼 상태(t = −2.21, *p* = .028), 최종학력(F = 4.18, *p* = .017), 임상경력(F = 3.57, *p* = .015), 직위(t = −2.58, *p* = .011)에서 유의한 차이가 나타났다. Scheffe 사후검정 결과, 임상경력 12∼35개월(3.89 ± 0.40점)이 72개월 이상 집단(3.60 ± 0.46점)에 비해 유의하게 높은 환자안전문화가 나타났다. ASP 참여의 경우 성별(t = −2.76, *p* = .006), 연령(t = −2.64, *p* = .009), 결혼 상태(t = −2.41, *p* = .017), 직위(t = −2.44, *p* = .016)에서 유의한 차이가 나타났다.

항생제 적정사용 관리 관련 특성에서 ASP 인식의 경우 ASP에 의한 항생제 사용 감소(t = 3.84, *p* < .001)와 ASP에 의한 환자 돌봄 및 안전 향상(t = 2.61, *p* = .010)에서 유의한 차이가 나타났다. 환자안전문화에서는 ASP에 의한 항생제 사용 감소(t = 2.85, *p* = .005)와 ASP에 의한 환자 돌봄 및 안전 향상(t = 2.66, *p* = .009)에서 유의한 차이가 나타났다. ASP 참여의 경우 ASP에 의한 항생제 사용 감소(t = 2.05, *p* = .042)에서 유의한 차이가 나타났다(Table 3).

### 4. 항생제 적정사용 관리 인식, 환자안전문화, 항생제 적정사용 관리 참여의 상관관계

ASP 인식은 환자안전문화(r = .41, *p* < .001)와 ASP 참여(r = .46, *p* < .001) 모두와 유의한 정적 상관관계를 나타냈다. 또한 환자안전문화는 ASP 참여(r = .38, *p* < .001)와도 유의한 정적 상관관계를 보였다(Table 4).

### 5. 항생제 적정사용 관리 참여 영향요인

ASP 참여에 영향을 미치는 요인을 확인하기 위해 모든 독립변수를 동시에 투입하는 입력(Enter) 방식을 사용하여 다중회귀분석을 실시하였다. ASP 참여와 유의한 차이를 보였던 일반적 특성 변수인 연령, 성별, 결혼여부, 직위와 ASP 관련 특성인 ASP에 의한 항생제 사용감소 여부를 독립변수로 투입하였다. 또한, 상관관계 분석 결과 ASP 참여와 유의한 정적 상관관계를 보인 ASP 인식과 환자안전문화를 주요 독립변수로 포함하였다. 이 중 성별, 결혼여부, 직위, ASP에 의한 항생제 사용감소 여부는 가변수(dummy variables) 처리하였다.

회귀분석의 기본 가정을 충족하는지 확인하기 위해 정규성, 다중공선성, 자기상관성 검증을 실시하였다. 정규성 검정 결과 왜도(skewness)와 첨도(kurtosis)의 절댓값이 모두 2 미만으로 나타나 정규성에 크게 벗어나지 않는 것으로 확인되었다. 독립변수 간 자기 상관성 여부를 확인하기 위한 Durbin-Watson 지수는 1.97로 2에 가까운 값으로 측정되어 자기 상관은 없는 것으로 나타났다. 다중공선성 여부를 확인한 결과 공차 한계(tolerance)는 .48∼.91로 .1 이상이었으며, 분산팽창요인(variation inflation factor, VIF)은 1.10∼2.10로 10 미만이었다. 일반적으로 공차 한계가 .1 이하이거나 VIF가 10 이상일 경우 다중공선성이 존재하는 것으로 간주되므로, 본 연구에서 다중공선성 문제는 없는 것으로 확인되었다.

다중회귀분석을 실시한 결과 ASP 참여에 영향을 미치는 요인으로 ASP 인식(β = .35, *p* < .001)과 환자안전문화(β = .18, *p* = .023)가 포함되었으며, ASP 참여에 대한 설명력은 25.0%로 나타났다(F = 8.36, *p* < .001) (Table 5).

## 논의

본 연구는 중환자실 간호사의 ASP 인식과 환자안전문화, ASP 참여 정도를 파악하고 ASP 참여에 영향을 미치는 요인을 규명하고자 시행되었으며 주요 연구 결과에 대해 다음과 같이 논의하고자 한다.

중환자실 간호사의 ASP 인식은 78.79점으로, 동일한 도구를 사용한 Mustafa 등[13]의 연구에서 보고한 90.00점 보다 낮게 나타났다. 또한 5점 척도 평균은 3.94점으로, 도구는 다르지만 국내 일반간호사를 대상으로 한 연구[20]의 4.42점보다 낮은 수준이었다. Mustafa 등[13]의 연구에서 더 높은 ASP 인식 중앙값을 보인 것은, 연구가 수행된 파키스탄의 ASP 관련 정책과 제도적 영향을 받은 결과로 해석될 수 있다. 파키스탄에서는 2014년 'Antibiotic Stewardship Initiative in Pakistan' 정책 발표 이후, 다양한 캠페인과 전문가 토론, ASP 주간 운영 등을 통해 적극적인 인식 제고 노력을 시행해 왔으며[13,29], 이러한 점이 간호사의 인식 수준 향상에 기여했을 가능성이 있다. 우리나라 또한 WHO의 글로벌 행동 계획에 따라 '제2차 국가 항생제 내성 관리 대책’을 수립하고, 상급종합병원과 종합병원을 중심으로 ASP 시범사업을 운영하는 등 제도적 기반을 마련해 왔다[4,30,31]. 그러나 이러한 정책적 노력에도 불구하고 본 연구에서 중환자실 간호사의 ASP 인식 수준은 상대적으로 낮은 편에 속한다. 세부 문항 결과에서도 항생제 처방의 합리성 촉진이나 의료 질 향상에 대한 인식은 높은 편이었지만 치료 비용 절감에 대한 인식이 상대적으로 낮았다는 점에서 간호사의 ASP 인식이 항생제 치료 비용 절감 등 실질적인 측면보다는 환자안전이나 의료 질 향상과 같은 가치 중심적 측면에 치우쳐 있음을 시사한다. 이는 현재 제도적 기반이 마련되어 시행되고 있으나, 현장 간호사들이 체감할 수 있는 역할 규명이나 교육, 참여 기회 등 실행 전략이 충분히 전달되지 않았기 때문으로 해석할 수 있다. 특히 중환자실은 항생제 사용량이 많고 내성균 발생 위험이 높은 부서임에도 불구하고, 간호사의 ASP 인식 수준이 높지 않다는 점은 정책적 노력과 간호 실무 간의 간극을 시사한다. 따라서 향후에는 제도적 기반과 더불어, 현장의 인식을 제고할 수 있는 실무자 대상 교육 강화, 다학제 협력 구조 구축, 간호사의 역할 명확화 등 실행 단계에서의 지속적인 지원이 요구된다.

ASP 참여는 60.67점으로 ASP 인식 수준에 비해 낮았다. 동일한 도구를 사용한 Mustafa 등[13]의 연구에서는 28.1점으로 보고되었다. 두 연구는 문항 수가 달라 직접 비교에 한계가 있고, 특히 Mustafa 등[13]의 도구에는 항생제 사용관리 위원회, 의료기관 항생제 치료 가이드라인 개발, 의료기관 내 항생제 종류의 관리 참여와 같은 항목이 포함되어 있었는데 이들은 전체 문항 중 참여도가 상대적으로 낮은 항목에 속하였다. 본 연구에서는 이러한 세 문항을 제외한 13문항으로 참여 정도를 측정하였기에 평균 점수가 상대적으로 높게 나타났을 것으로 해석된다. 그러나 두 연구 모두 간호사들의 높은 인식 수준에도 불구하고 실제 참여는 낮게 나타났다는 점에서 공통점을 보였다. 이는 간호사들이 ASP의 중요성을 인식하고 있음에도 불구하고, 임상 현장에서의 실질적인 참여로 이어지지 못하고 있음을 시사한다. 기존 연구에서는 ASP에 대한 낮은 인식이 간호사의 ASP 참여를 저해하는 주요 요인으로 보고하고 있지만[13,32], 본 연구와 Mustafa 등[13]의 연구에서 나타난 결과는 간호사들의 ASP 인식이 높음에도 불구하고 실제 참여 정도는 낮게 나타났다는 점은 주목할 필요가 있다. 세부 문항 결과, 항생제 부작용에 대한 논의나 처방된 항생제에 대한 검토와 같은 일상적인 간호사의 업무 영역에서는 비교적 활발하게 참여하고 있었으나, 중환자의 항생제 치료 결정 참여나 항생제 소비량 평가 등 적극적이고 전문적인 ASP 역할 수행에서는 낮은 참여 정도를 보였다. 이는 간호사의 높은 인식이 실제 참여로 이어지기 위해서는 개인적 요인 뿐만 아니라 명확한 역할 규명과 같은 환경적 요인이 중요함을 시사한다[7-9,14-16]. 특히, 현재 국내에서 개발, 보급된 지침에서는 ASP 운영 필수인력으로 의사와 약사만 명시되어 있고, 간호사의 역할이 구체적으로 제시되지 않아 간호사의 참여를 제한하는 요인이 될 수 있다. 지침에는 간호사가 환자 치료의 소통과 업무 흐름, 교육에 있어 중요한 역할을 수행하는 등 ASP 운영에 반드시 포함되어야 한다고 권고하고 있으나[10,30], 간호사의 구체적인 역할이 명확히 규정되지 않아 실질적인 참여가 제한되는 것이 현실이다. 따라서 향후 ASP 지침에서는 간호사의 역할과 책임을 명확히 하고, ASP 전문인력으로 공식적으로 포함시키는 방안이 필요하다. 또한 간호사를 대상 ASP 전문 교육과정을 체계적으로 개발하여 간호사의 실제 ASP 참여를 높이는 것이 중요하다.

환자안전문화 정도는 5점 만점에 3.66점으로 동일한 도구를 사용한 중환자실 간호사의 환자안전문화 3.55점[33] 보다는 약간 높았으나, 일반병동 간호사의 환자안전문화 3.84점[34] 보다는 낮았다. 간호사의 환자안전문화를 저하시키는 요인으로 과도한 업무 부담, 스트레스가 많은 환경, 비효율적인 의사소통 등이 제시되고 있는데[35], 이중 비효율적 의사소통은 환자안전문화 뿐만 아니라 ASP 참여를 저하시키는 요인으로도 보고되고 있다[9,15,35]. 중환자실에서 간호사가 의사와의 의사소통에서 원활하게 정보를 교환하지 못하면 환자안전문화에 부정적인 영향을 미치며[35], ASP 역시 다학제 협력과 의사소통이 필수적이므로 조직 내에서 개방적이고 협력적인 의사소통 환경을 조성하는 것이 중요한 과제로 제시된다[15,35]. 또한 중환자실 구성원들과 함께 성숙한 환자안전문화 형성을 위한 노력과 환자안전문화 교육을 체계적으로 보완하는 것이 필요하다.

대상자의 일반적 특성에 따른 ASP 참여를 살펴보았을 때, 임상경력 및 중환자실 경력이 많을수록 ASP 참여 수준이 높았으나 유의한 수준에는 미치지 못하였다. 이러한 결과는 임상경력과 ASP 참여 사이에 정적 상관관계가 있다는 선행연구의 결과와 차이가 있다[7,8]. 이는 본 연구의 대상자가 중환자실 간호사인 것에 반해 일반병동 간호사가 포함되어 있고, 중환자실 간호사의 항생제 내성과 항생제 사용관리에 대한 지식과 태도가 일반병동 및 기타부서 간호사에 비해 유의하게 높았다는 선행연구[28]와 관련이 있어 보인다. 또한 연령과 ASP 참여 간에 정적 상관관계가 있다고 나타났다. Mustafa 등[13]은 ASP 참여가 간호사의 연령에 따라 유의한 차이가 있다고 하여 본 연구 결과를 지지하고 있지만, Nie 등[36]의 연구에서는 연령이 ASP 참여와 유의한 상관관계나 영향요인으로 나타나지 않아 차이가 있다. 다만, 일반적으로 연령이 높을수록 항생제 사용관리 역량이 축적되어 ASP 참여가 높아지는 것으로 해석해 볼 수 있겠다.

중환자실 간호사의 ASP 참여에 영향을 미치는 요인은 ASP 인식과 환자안전문화로 나타났으며, ASP 참여와 상관관계를 보인 연령은 최종 모델에서 제외되었다. 특히, ASP 인식은 중환자실 간호사의 ASP 참여에 영향을 미치는 주된 요인으로 나타나 간호사의 인식 수준이 높을수록 실제 ASP에 참여하는데 도움이 된다는 선행연구를 지지하였다[34,37]. 본 연구에서 ASP 인식의 세부 문항 중 ‘항생제의 합리적 처방 촉진’과 ‘의료 질 향상’에 대한 인식 수준이 높았던 점을 고려할 때, 이러한 간호사의 가치중심적 인식을 실제 ASP 참여로 연결할 수 있는 전략이 필요하다. 중환자실 간호사들은 ‘항생제 부작용 논의’, ‘처방 오류에 대한 피드백’과 같은 기본적인 협력 활동에는비교적 적극적으로 참여하고 있었다. 반면, ‘병원 내 항생제 사용량 정보 확인’이나 ‘항생제 치료 기간 모니터링 및 중단 논의’와 같은 보다 전문적이고 체계적인 역할을 요구하는 영역에서는 참여도가 낮았다. 이는 간호사가 ASP의 다학제적 협력에서 보다 적극적으로 역할을 수행하기 위해서는 구체적인 ASP 실무 중심의 역할을 규명하고 이를 실질적으로 지원하는 체계와 교육이 마련되어야 함을 시사한다. 따라서 의료기관 간호사를 대상으로 하는 전문적인 ASP 교육 프로그램 개발이 요구되며[7-9,12-16,19-22], 이를 효과적으로 시행하기 위해서는 기존 감염관리 교육과 차별화되어야 한다[22]. 기존의 감염관리 교육이 주로 표준주의 준수나 다제내성균 전파 예방 등 일반적인 감염관리 방법에 초점을 맞추었다면 ASP 교육은 항생제 내성 문제와 ASP의 중요성, ASP에서 간호사의 실무 역할, 환자 및 보호자 교육, 다학제적 협력 등에 중점을 두어야 한다. 이를 위해서는 항생제 오남용, 항생제 내성 발생 기전, 항생제 사용관리 방법, 그리고 ASP에서 간호사의 역할 인식 수준을 사전에 평가하고 이해하는 과정이 필요하다[32]. 또한 이러한 ASP 교육은 학부 과정에서부터 이루어져야 한다는 국제적 논의가 점차 강화되고 있다[22,38,39]. 영국의 경우 간호학부 과정의 63.2%에서 ASP 교육과정을 필수적으로 포함하고 있고[40], 뉴질랜드 정부 역시 간호학부 과정에서 간호사의 ASP에서 역할과 책임을 교육하도록 강조하고 있다[41]. 그러나 현재 국내의 ASP 정책은 간호사나 간호학부 학생을 대상으로 한 교육과정이 포함되어 있지 않아 향후 정책적 개선이 필요한 부분이다[19]. 따라서 간호학부 과정에서는 미래 간호사들이 ASP의 개념과 역할을 교육하고, 의료기관에서는 실무 간호사를 대상으로 한 지속적인 ASP 교육 프로그램을 운영하여, 이를 의료기관의 ASP 중재와 연계한다면 간호사의 ASP 인식을 향상시키고 결과적으로 ASP 참여를 유도할 수 있을 것으로 기대된다.

환자안전문화 역시 ASP 참여에 유의한 영향을 미치는 것으로 나타났으며, 이는 낮은 환자안전문화 수준이 ASP 참여에 장애요인이 될 수 있다는 선행연구를 지지하였다[7-9,25]. 또한, 본 연구 결과는 환자안전문화 수준이 높을수록 손위생[42], 표준주의[43] 수행도가 향상된다는 선행연구와 일맥상통하며 이는 항생제 내성 관리를 위한 핵심 전략인 ASP 참여 및 감염관리 수행 촉진에 있어 긍정적인 환자안전문화 조성이 선행되어야 함을 시사한다. 이러한 맥락에서 국가 항생제 내성 관리 대책이 항생제 내성균 발생을 감소시키는 ASP와 확산 방지를 위한 감염관리를 주요 전략으로 제시하고 있음을 고려할 때[4] 조직 내 긍정적인 환자안전문화 조성을 통해 두 가지 핵심 전략의 효과적인 이행을 도모할 수 있다. 따라서 실무 현장에서 환자안전문화를 정착시키기 위해서는 명확한 의사소통 채널과 구조화된 보고 체계를 구축하여 의료진 간 협력을 증진시키고 간호사를 비롯한 의료진을 대상으로 효과적인 의사소통 및 팀워크 함양을 목표로 하는 체계적이고 지속적인 교육 프로그램 운영이 필요하다[35]. 또한 리더십의 적극적인 개입은 ASP 운영과 환자안전문화 정착 모두에서 핵심적인 역할을 하므로 명확한 목표 설정과 자원 지원을 통해 간호사의 참여를 유도하고[35] 궁극적으로 항생제 사용관리의 질 향상과 환자안전 수준 제고에 기여할 수 있을 것으로 판단된다.

본 연구는 국내 중환자실 간호사의 ASP 인식과 참여, 환자안전문화 정도를 파악하고 ASP 참여에 영향을 미치는 요인을 조사한 연구로 개인적 측면으로 ASP 인식이, 조직적 측면으로 환자안전문화가 ASP 참여에 영향을 미치는 것을 확인하였다. 본 연구를 통해 ASP 참여에 영향을 미치는 개인적 요인과 조직적 요인을 통합적으로 탐색하였다는 측면에서 의의가 있다.

본 연구는 대구광역시에 소재한 2개 상급종합병원 중환자실 간호사를 대상으로 편의표집하여 자료를 수집하였기 때문에 연구결과를 전체 중환자실 간호사에게 일반화하는데 한계가 있다. 또한 본 연구는 국내 ASP 시법사업이 본격적으로 확대되기 이전 시점에 수행되었으며, 당시 중환자실 간호사를 대상으로 한 ASP 관련 교육이 시행되지 않았고 간호사의 역할을 규명하거나 이를 지원할 제도적 체계 및 가이드라인도 마련되어 있지 않았다. 이에 따라 연구에 참여한 간호사들이 ASP 관련 정보에 노출될 기회가 제한적이었고, 이는 ASP 인식 및 참여 수준에도 영향을 미쳤을 가능성이 있다. 아울러, 일반병동 간호사와는 ASP에 대한 인식이나 참여 수준에서 차이가 있을 가능성이 있다. 또한 국내 간호사를 대상으로 한 ASP 관련 연구가 부족하여 본 연구 결과를 선행연구와 직접 비교하는 데에도 제한점이 있다. 본 연구에서 ASP 참여의 영향요인으로 ASP 인식과 환자안전문화의 설명력은 25.0%로 확인되어 추후 연구를 통해 ASP 참여에 영향을 미치는 다양한 변수를 규명하는 노력이 필요하다.

## 결론

본 연구는 중환자실 간호사를 대상으로 ASP 참여에 영향을 미치는 요인을 파악하여 간호사의 ASP 참여를 높이기 위한 구체적인 전략 마련의 근거자료를 제공하고자 시도되었다. 연구 결과 개인적 요인으로 ASP 인식이, 조직적 요인으로 환자안전문화가 ASP 참여에 영향을 미치는 것을 확인하였다. 따라서 중환자실 간호사의 ASP 참여를 높이기 위해서는 ASP 인식 제고를 위한 지속적 교육과 함께 조직적 차원에서 환자안전문화를 확산하여 환자안전에 대한 인식을 고양시키는 것이 필요하다.

본 연구 결과를 바탕으로 다음과 같이 제언하고자 한다.

첫째, 본 연구는 일 지역 두 개의 상급종합병원 중환자실 간호사만을 대상으로 하였으므로, 추후 지역 및 의료기관 유형을 고려하여 대상자를 확대하고 다양성을 확보하는 반복 연구를 제언한다.

둘째, ASP 참여에 영향을 미치는 다양한 차원의 요인을 탐색하기 위한 심층 연구를 제언한다.

셋째, 국내 의료기관별 상황과 특성을 반영한 ASP 내 간호사의 역할 규명 이를 뒷받침할 수 있는 제도적 지원체계 및 가이드라인 마련을 제언한다.

넷째, 임상 현장 적용 중심의 체계적이고 지속적인 ASP 교육 프로그램을 개발하고 그 효과를 검정하는 연구를 제언한다.

## Figures and Tables

**Table 1.** General and Antimicrobial Stewardship Program-related Characteristics (N = 159)

Characteristics	Categories	n (%)	M ± SD
Sex	Men	20 (12.6)	
	Women	139 (87.4)	
Age (years)	< 30	96 (60.4)	29.57 ± 4.68
	≥ 30	63 (39.6)	
Marital status	Single	121 (76.1)	
	Married	38 (23.9)	
Educational level	Associate’s degree	18 (11.3)	
	Bachelor’s degree	130 (81.8)	
	Master’s degree	11 (6.9)	
Clinical experience (months)	12∼35	16 (10.1)	77.40 ± 52.80
	36∼71	65 (40.9)	
	≥ 72	78 (49.0)	
ICU experience (months)	12∼35	36 (22.6)	54.00 ± 32.80
	36∼71	81 (51.0)	
	≥ 72	42 (26.4)	
Department	Medical ICU	89 (56.0)	
	Surgical ICU	70 (44.0)	
Current position	Staff	152 (95.6)	
	Charge nurse	7 (4.4)	
Antimicrobial resistance is a problem	Yes	159 (100.0)	
	No	0 (0.0)	
ASPs reduce antimicrobial use	Yes	95 (59.7)	
	No	64 (40.3)	
ASPs promote patient safety	Yes	143 (89.9)	
	No	16 (10.1)	

M = Mean; SD = Standard deviation; ICU = Intensive care unit; ASP = Antimicrobial stewardship program.

**Table 2.** Levels of Perceptions and Involvement Regarding Antimicrobial Stewardship Programs, and Patient Safety Culture (N = 159)

Variables	M ± SD (100-point scale)	M ± SD (1~5 scale)	Min	Max
Perceptions of ASP	78.79 ± 12.43	3.94 ± 0.62	2.00	5.00
ASPs promote reasonable prescription of antimicrobials		4.03 ± 0.63	1.00	4.00
ASPs help control antimicrobial resistance		3.95 ± 0.68	1.00	4.00
ASPs reduce the overuse of antimicrobials		3.91 ± 0.72	1.00	4.00
ASPs reduce the cost of treatment		3.81 ± 0.76	1.00	4.00
ASPs improve medical quality		4.00 ± 0.72	1.00	4.00
Patient safety culture (subscale)		3.66 ± 0.42	2.40	4.89
Leadership (9 items)		3.66 ± 0.64	1.56	5.00
Teamwork (6 items)		3.84 ± 0.48	2.17	4.83
Patient safety knowledge and attitude (5 items)		4.11 ± 0.46	3.00	5.00
Patient safety policies and procedures (4 items)		3.47 ± 0.62	1.75	5.00
Non-discriminatory environment (4 items)		3.56 ± 0.64	2.00	5.00
Patient safety improvement system (4 items)		3.37 ± 0.59	1.75	5.00
Patient safety priorities (3 items)		3.34 ± 0.70	1.00	5.00
Involvement in the ASP	60.67 ± 11.61	3.03 ± 0.58	1.15	4.69
I review prescriptions of antimicrobials		3.85 ± 0.80	1.00	5.00
I proactively intervene in problematic prescriptions of antimicrobials		2.99 ± 0.89	1.00	5.00
I comment on antimicrobial prescriptions and medical orders		3.33 ± 0.96	1.00	5.00
I provide feedback on antimicrobial prescriptions and medical orders		3.55 ± 0.88	1.00	5.00
I participate in decision-making of antimicrobial treatment for critically ill patients		2.03 ± 0.92	1.00	5.00
I provide advice to doctors regarding the intravenous-to-oral conversion of antimicrobials		2.23 ± 0.99	1.00	5.00
I provide advice to doctors about the adverse drug effects of antimicrobials		3.91 ± 0.84	2.00	5.00
I provide advice to doctors about drug interactions related to antimicrobials		3.19 ± 0.98	1.00	5.00
I monitor the duration of antimicrobial treatment and recommend appropriate cessation		2.38 ± 0.93	1.00	5.00
I provide feedback to doctors on bacterial resistance		3.33 ± 1.04	1.00	5.00
I monitor and evaluate antimicrobial consumption in hospital		2.16 ± 0.93	1.00	4.00
I communicate with doctors on anti-infective treatment		2.97 ± 0.91	1.00	5.00
I communicate with patients and family members on antimicrobial treatment		3.50 ± 0.86	1.00	5.00

M = Mean; SD = Standard deviation; Min = Minimum; Max = Maximum; ASP = Antimicrobial stewardship program.

**Table 3.** Differences in Variables by General and Antimicrobial Stewardship Program-related Characteristics (N = 159)

Characteristics	Categories	Perceptions of ASP		Patient safety culture		Involvement of ASP	
		M ± SD	t/F (*p*)	M ± SD	t/F (*p*)	M ± SD	t/F (*p*)
			Scheffé		Scheffé		Scheffé
Sex	Men	4.17 ± 0.68	−1.79 (.076)	3.91 ± 0.48	2.97 (.003)	3.36 ± 0.62	−2.76 (.006)
	Women	3.91 ± 0.61		3.62 ± 0.40		2.99 ± 0.56	
Age (years)	< 30	3.87 ± 0.52	−1.62 (.109)	3.66 ± 0.41	−0.10 (.920)	2.94 ± 0.56	−2.64 (.009)
	≥ 30	4.04 ± 0.74		3.66 ± 0.44		3.18 ± 0.58	
Marital status	Single	3.86 ± 0.61	−2.84 (.003)	3.62 ± 0.39	−2.21 (.028)	2.97 ± 0.56	−2.41 (.017)
	Married	4.18 ± 0.61		3.79 ± 0.48		3.23 ± 0.60	
Educational level	Associate’s degree	3.81 ± 0.74	0.49 (.613)	3.86 ± 0.44	4.18 (.017)	3.10 ± 0.38	1.65 (.196)
	Bachelor’s degree	3.95 ± 0.55		3.62 ± 0.39		3.00 ± 0.60	
	Master’s degree	4.02 ± 1.09		3.86 ± 0.56		3.31 ± 0.52	
Clinical experience (months)	12∼35^a^	3.83 ± 0.52	1.05 (.354)	3.89 ± 0.40	3.57 (.015)	3.00 ± 0.59	0.51 (.599)
	36∼71^b^	3.88 ± 0.52		3.68 ± 0.34	c < a	2.98 ± 0.49	
	≥ 72^c^	4.01 ± 0.71		3.60 ± 0.46		3.08 ± 0.65	
ICU experience (months)	12∼35^a^	3.96 ± 0.55	0.03 (.971)	3.77 ± 0.48	3.64 (.029)	2.91 ± 0.57	1.54 (.218)
	36∼71^b^	3.94 ± 0.60		3.68 ± 0.39	c < a	3.03 ± 0.56	
	≥ 72^c^	3.92 ± 0.69		3.53 ± 0.41		3.09 ± 0.60	
Department	Medical ICU	3.91 ± 0.64	−0.78 (.438)	3.69 ± 0.46	0.83 (.410)	2.99 ± 0.58	−1.03 (.305)
	Surgical ICU	3.98 ± 0.60		3.63 ± 0.36		3.09 ± 0.58	
Position	Staff	3.93 ± 0.61	−1.26 (.209)	3.64 ± 0.41	−2.58 (.011)	3.01 ± 0.56	−2.44 (.016)
	Charge nurse	4.23 ± 0.83		4.05 ± 0.55		3.55 ± 0.78	
ASPs reduce antimicrobial use	Yes	4.09 ± 0.63	3.84 (< .001)	3.74 ± 0.42	2.85 (.005)	3.11 ± 0.57	2.05 (.042)
	No	3.72 ± 0.54		3.55 ± 0.40		2.92 ± 0.58	
ASPs promote patient safety	Yes	3.98 ± 0.63	2.61 (.010)	3.69 ± 0.41	2.66 (.009)	3.03 ± 0.57	−0.21 (.833)
	No	3.56 ± 0.43		3.40 ± 0.41		3.06 ± 0.68	

M = Mean; SD = Standard deviation; ICU = Intensive care unit; ASP = Antimicrobial stewardship program.

**Table 4.** Correlations among Variables (N = 159)

Variables	1	2	3
	r (*p*)		
1. Perceptions of ASP	1	-	-
2. Patient safety culture	.41 (< .001)	1	-
3. Involvement in the ASP	.46 (< .001)	.38 (< .001)	1

ASP = Antimicrobial stewardship program.

**Table 5.** Factors Influencing Involvement in the Antimicrobial Stewardship Program (N = 159)

Variables	B	SE	β	t	*p*
(Constant)	1.02	0.71		1.43	.155
Sex (women)^†^	0.21	0.13	.12	1.64	.101
Age	0.00	0.01	.02	0.16	.875
Marital status (single)^‡^	0.04	0.12	.03	0.36	.723
Position (charge nurse)^§^	−0.29	0.24	−.10	−1.19	.236
ASP reduces antimicrobial use (yes)^∥^	−0.02	0.09	−.02	−0.23	.820
Perceptions of ASP	0.33	0.07	.35	4.45	< .001
Patient safety culture	0.25	0.11	.18	2.25	.023
R^2^ = .28, Adjusted R^2^ = .25, F = 8.36, *p* < .001					

SE = Standard error; ASP = Antimicrobial stewardship program.

^†^Sex (reference = men); ^‡^Marital status (reference = married); ^§^Position (reference = staff); ^∥^ASP reduces antimicrobial use (reference = no).

## Data Availability

Please contact the corresponding author for data availability.
